# Nesplora Ice Cream test: a normative study of a virtual reality-based executive function assessment in adults

**DOI:** 10.3389/fpsyg.2025.1561802

**Published:** 2025-03-31

**Authors:** Fidel Rebón-Ortiz, Débora Areces, Miguel Saura-Carrasco, Claudia Flores, Celestino Rodríguez, Unai Díaz-Orueta, Gema Climent, Irene Alice Chicchi Giglioli

**Affiliations:** ^1^Giunti-Nesplora SL, Madrid, Spain; ^2^Department of Psychology, International University of La Rioja (UNIR), Logroño, Spain; ^3^Faculty of Psychology, University of Oviedo, Oviedo, Spain; ^4^Department of Psychology, Maynooth University, Maynooth, Ireland; ^5^Department of Psychology, University of Almeria, Almería, Spain

**Keywords:** neuropsychological assessment, virtual reality, executive functions, ecological validity, normative data

## Abstract

This study aims to establish normative data for participants aged 17 to 80 who completed the Nesplora Ice Cream test, a virtual reality tool designed to assess executive functions. The objective is to provide a comprehensive reference for evaluating executive function performance in healthy adults across different age groups. A total of 419 participants (51% female) were recruited from nine locations in Spain. Trained evaluators administered the Nesplora Ice Cream test. The study utilized empirical analysis to identify key factors related to executive function, focusing on planning, learning, and flexibility. Cluster analysis was employed to define age groups for each factor: 17–40, 41–61, and 62–80 for planning; 17–44, 45–61, and 62–80 for learning; and 17–20, 21–36, and 37–80 for flexibility. The analysis revealed three main factors—planning, learning, and flexibility—that characterize executive function performance. No significant gender differences were found. Descriptive normative data were provided based on age and gender. Confirmatory factor analysis supported the three-factor structure of the test. Additionally, data on the validity, reliability, and internal consistency of the test were included. These normative data are valuable for assessing executive functions in an ecologically valid way. The findings provide a robust reference point for studying the early identification of executive dysfunction in adults and the impact of neurodegenerative conditions in clinical settings. Further research is needed to evaluate the test’s sensitivity and specificity in clinical populations. These norms enable the development of timely, personalized interventions for individuals showing executive function impairments.

## 1 Introduction

Executive functions (EFs) are a set of high-level cognitive processes essential for goal-directed behavior, including planning, decision-making, problem-solving, and self-regulation (Miyake et al., 2000). These functions are crucial for everyday tasks, enabling individuals to manage complex activities, adapt to changing situations, and maintain mental flexibility (Friedman and Miyake, 2004). As an individual age, EFs undergo significant changes throughout the lifespan, with distinct patterns observed across different life stages. In early childhood, executive functions are still developing, with attention, working memory, and cognitive flexibility gradually improving as the brain matures (Diamond, 2013; Ferguson et al., 2021). During adolescence, there is a marked improvement in the ability to plan, organize, and control impulses, as the prefrontal cortex undergoes substantial growth and refinement (Luna et al., 2004). These improvements in EF are critical for managing complex tasks, academic challenges, and social interactions. As a result, in adulthood, executive functions continue to be refined, supporting the capacity to multitask, make decisions, and adjust to changing circumstances (Salthouse, 2010). As individuals move into middle and later adulthood, changes in EFs reflect the ongoing adaptation to life demands, with some cognitive functions becoming more efficient in certain contexts, such as increased reliance on experience and accumulated knowledge (Park and Reuter-Lorenz, 2009). Throughout the lifespan, the development and maintenance of EFs are influenced by various factors, including genetics, environment, and life experiences, highlighting the dynamic nature of cognitive processes as individuals navigate different stages of life (Park and Reuter-Lorenz, 2009).

Deficits in executive functions are commonly observed in individuals with neurological conditions, significantly impacting their daily functioning and quality of life. Impairments in EFs are prevalent in disorders such as traumatic brain injury (TBI), attention-deficit/hyperactivity disorder (ADHD), Alzheimer’s disease, Parkinson’s disease, and schizophrenia (Goldstein et al., 2014; Diamond, 2013). These deficits manifest in difficulties with problem-solving, impulse control, and cognitive flexibility, often leading to challenges in independent living, employment, and social interactions (Anderson et al., 2010). Understanding and assessing EF impairments in these populations are crucial for developing effective interventions and improving patient outcomes.

Over the past few decades, the assessment of EFs has been predominantly conducted using traditional neuropsychological tests, which, while effective, have limitations in terms of ecological validity and engagement (Burgess et al., 1998). These tests often fail to capture the complexity of real-world cognitive challenges that individuals face daily, as they are typically designed to assess isolated cognitive abilities in controlled, artificial settings (Howieson, 2019). Executive function assessments using traditional methods, such as the Wisconsin Card Sorting Test (WCST) or the Stroop Test, often focus on tasks like cognitive flexibility, inhibitory control, and working memory in situations that do not fully represent the dynamic and context-rich nature of daily life (Lezak et al., 2012). While these tests provide valuable insights into basic cognitive processes, they do not fully account for how individuals interact with complex, ever-changing environments that require adaptive and context-sensitive decision-making (Lezak et al., 2012).

The limitations of traditional assessments have led to a growing interest in innovative approaches, with virtual reality (VR) emerging as a promising tool for assessing executive functions. VR allows for the creation of immersive, interactive environments that simulate real-life situations in ways that traditional paper-and-pencil tests cannot. In contrast to static tests, VR provides a dynamic and multifaceted platform for measuring EFs, capturing behaviors and cognitive processes that occur in more ecologically valid contexts. These VR environments can involve tasks requiring problem-solving, multitasking, planning, and decision-making, thus offering a more comprehensive evaluation of cognitive flexibility, working memory, and self-regulation (Rizzo and Koenig, 2017). Numerous reviews have explored the use of VR in neuropsychological assessments, demonstrating its potential advantages in accurately measuring executive functions in both clinical and non-clinical populations (Borgnis et al., 2022; Kirkham et al., 2024). Borgnis et al. (2022) analyzed 301 articles, demonstrating that VR-based tools offer promising solutions for ecological assessment and treatment of EFs in both healthy subjects and various clinical populations. Another systematic review by Kirkham et al. (2024) focused specifically on immersive VR assessments of EF, identifying 19 studies that covered various EF components such as inhibitory control, cognitive flexibility, working memory, planning, and attention. These reviews underscore the potential of VR to overcome the ecological limitations of traditional neuropsychological tests and increase test sensitivity and ecological validity in EF assessment.

According to these reviews, VR-based assessments of executive functions have several advantages. First, they allow for the creation of complex, context-rich scenarios where individuals can be tested in environments that mirror real-life situations, such as driving, navigating virtual spaces, or interacting in social settings (Adriasola et al., 2024; Cañada et al., 2024). This allows clinicians to observe how individuals perform in tasks requiring planning, attention, impulse control, and flexibility under various conditions, such as time pressure or emotional stress. Secondly, VR enables the use of adaptive scenarios that change according to the individual’s performance, providing a more personalized and precise assessment of their executive functioning abilities (Burgess et al., 1998). Additionally, research has highlighted the convergent validity of VR-based assessments with traditional neuropsychological tests, demonstrating that these tools can accurately measure EF constructs while improving engagement and ecological validity (Lee et al., 2024).

As research into VR applications for neuropsychological assessments continues to grow, studies have demonstrated that VR-based evaluations of EFs are not only feasible but also offer superior engagement and motivational advantages over traditional testing methods (Pieri et al., 2023). By presenting assessments that are more engaging and reflective of real-world challenges, VR can improve the accuracy and relevance of executive function evaluations, leading to better outcomes in both clinical and research settings.

Starting from these premises, Nesplora Ice Cream test (Climent and Tirapu, 2022) aims to bridge this gap by developing a normative framework for the use of VR in adult executive function assessment. Indeed, this study explores the feasibility and effectiveness of using VR-based tools to evaluate executive functions such as working memory, cognitive flexibility, inhibition, and attention in a diverse adult population. By establishing a comprehensive set of normative data, the Nesplora Ice Cream test intends to provide a robust, standardized tool for clinicians and researchers, facilitating the identification of executive function impairments and advancing our understanding of how executive functions operate across the lifespan (Diamond, 2013; Zelazo and Carlson, 2012).

Importantly, the Nesplora Ice Cream test has been previously established for children, providing a solid foundation for its expansion into adult populations (Fernandez et al., 2023). The rationale for adapting this test to adults is based on the need for ecologically valid assessments of executive functions that go beyond traditional methods. Given the increasing demand for tools that provide dynamic and contextually relevant evaluations, the Nesplora Ice Cream test presents an opportunity to improve the assessment of executive functions in adults, ensuring that cognitive challenges across the lifespan, including those in clinical populations, are better understood and addressed.

## 2 Materials and methods

### 2.1 Participants

The normative sample consisted of 419 participants (51% female), aged 17 to 80 years, recruited across nine testing sites in Spain: San Sebastián, Bilbao, Murcia, Valencia, Galicia, Ávila, Granada, Oviedo, and Madrid. Inclusion criteria required Spanish proficiency and excluded neurological pathology, sensory alterations, or conditions that could limit virtual reality use. Consistent with previous studies (Climent et al., 2024; Iriarte et al., 2016), participants had no diagnosed psychiatric, neurodevelopmental, or neurological conditions. This approach aimed to reflect the general population accurately, allowing the prevalence of such conditions to mirror their natural occurrence.

The target participant number was determined to ensure representativeness of Spain’s general population based on age and gender, with sample size estimates derived from the 2016 census data and a cost–benefit analysis (Herranz and Prieto, 2005), recommending at least 400 participants with these two representative sociodemographic characteristics. The focus on age and sex is grounded in two key factors: (1) age and sex influence cognitive development and decline (Levine et al., 2021); (2) preliminary normative studies focused on age and sex (Mitrushina et al., 2005).

Informed consent forms were signed by participants or, for those aged 17, by their parents or legal guardians, as required by Spanish law. The study was approved by the Ethics Committee for Research with Human Beings at the University of the Basque Country (UPV-EHU), Spain, and adhered to the World Medical Association’s Declaration of Helsinki for human participant studies.

### 2.2 Materials

Nesplora Ice Cream is engineered to operate with commercially available virtual reality head-mounted displays (HMDs), specifically the Meta Quest 2, 3, and 3S models. The program provides support for these devices, thereby ensuring consistency across distinct models. Nevertheless, it is currently incompatible with VR HMDs from other manufacturers or preceding generations of commercial VR headsets. This constraint serves to uphold standardisation in the administration of tests and performance within varied clinical environments. Developed using the Unity engine, Nesplora Ice Cream precludes direct editing or modification to safeguard standardisation and validity. However, Nesplora engages in collaborations with researchers for validation studies, leveraging licenses, hardware, or expertise. Clinicians and researchers can conveniently establish the system on a standard PC or Mac, equipped with 4GB RAM and an Intel HD Graphics card or superior, in conjunction with the supported Meta Quest HMD and wired headphones.

### 2.3 Measures

#### 2.3.1 Nesplora Ice Cream test: tasks and metrics overview

**Figure 1:**
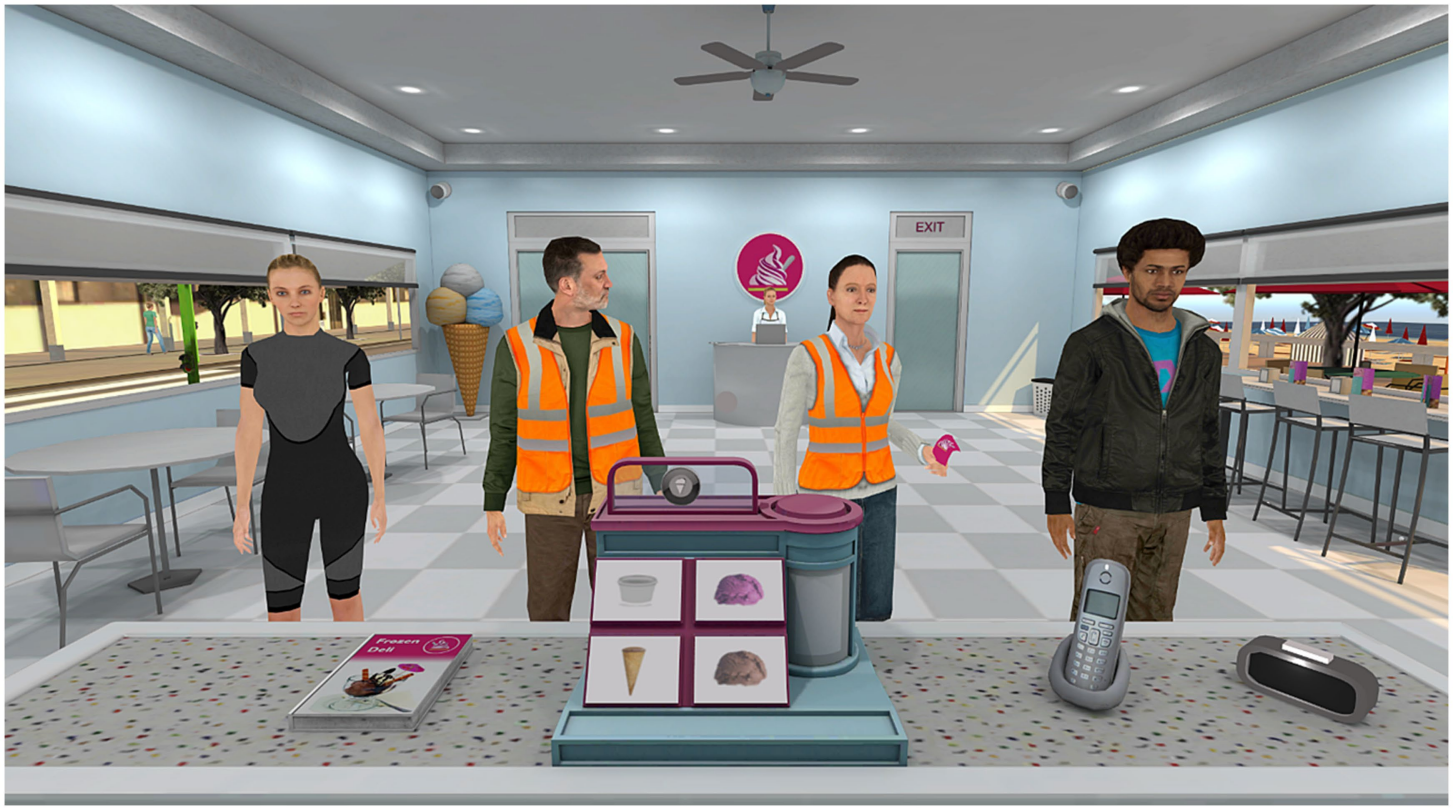
The VR environment of Nesplora Ice Cream test.

The Nesplora Ice Cream Test measures executive functions like planning, learning and cognitive flexibility through VR technology (Figure 1). Participants manage an ice cream shop with VR headsets and controllers, following audio instructions to organize orders and prepare ice creams while adapting to criteria changes. This setting appeared adequate to evaluate executive functions by simulating a real-world goal-oriented environment that requires multitasking, decision-making, and problem-solving. Managing customer orders, selecting the correct ingredients, and handling time constraints engage key executive functions such as planning, working memory and cognitive flexibility. Additionally, the familiar and engaging nature of an ice cream shop enhances motivation and ecological validity, making the assessment more reflective of daily cognitive demands. The test includes two evaluation tasks (A and B) after a familiarization and training phase with the VR system.

During familiarization, participants explore the ice cream shop and interact with elements like the ice cream machine and recipe book. In the training phase, the participant first receives audio instructions from the boss regarding the criteria they must follow when serving the customers who enter the ice cream shop. Once the correct service turns are assigned, each customer tells the participant which ice cream they want. The ice creams are shown in the recipe book, which the participant must learn to make the service faster. If the participant forgets an ice cream, they can still open the recipe book at any time. Similarly, if they forget the customer priority criteria, when no customers are in the shop, the participant can call the boss on the phone to have them. After training, the initial evaluation task (A) is started, which includes seven turns with four customers each, requesting a cumulative total of 28 ice creams. The four customers are visually distinct form one another, ensuring clear differentiation. In the subsequent evaluation task (B), the pre-established customers and criteria remain unchanged, while the four types of ice cream change. The number of turns and ice cream remains in agreement with those of the first evaluation task. Prevalent throughout these tasks, the test scrutinises fundamental executive functions: (a) Planning: This refers to the allocation of customer turns based on criteria defined by the supervisor. It is quantitatively measured by the precision of these allocations, which include correct assignments, erroneous allocations, and the temporal duration allocated to planning; (b) Learning correlated to working memory (task A): This is assessed by the precision with which to serve ice cream. The metrics encompass correct services, frequency of reference consultations, net correct responses (services performed without reference consultation), and the overall service duration; and (c) learning related to cognitive flexibility (task B): This involves the service of modified ice creams, unlike Task A. It is evaluated through the ability to adapt to new ice cream serving regulations, with metrics comprising performance accuracy, processing speed, persistence interference, and switching ability. The test uses a variety of performance metrics that span these tasks, providing a comprehensive evaluation of executive functions, with the main metrics presented in Table 1.

**Table 1:** Overview of metrics and variables in the Nesplora Ice Cream VR test.

Executive function	Metric	Metric description	Variable
Planning	Processing speed.	Measured by the time taken to correctly assign the order to customers and deliver the ice cream correctly.	Number of shifts correctly assigned in Part 1. Number of shifts correctly assigned in Part 2.
	Rule earning.	Evaluated by the accuracy in assigning the order and delivering the ice cream.	Learning potential to identify whether the customer wears a neoprene suit or not, (measured at Round 13). Learning potential when it comes to assigning the right order to the customers.
Learning – working memory (A)	Processing speed.	Measured during the preparation and delivery of the ice cream.	The number of total correct ice creams delivered correctly without looking at the recipe book on Part 1 rounds. Number of correct #1 ice creams delivered without looking at the recipe book in Part 1 rounds.
	Learning potential.	Evaluated based on the ability to learn how to serve ice cream correctly with minimal reference to the recipe book.	Learning potential in relation to making ice cream #1 correctly.
Learning – cognitive flexibility (B)	Processing speed.	Measured during the modification and adaptation to the new ice creams.	Number of total correct ice creams delivered correctly without looking at the recipe book on Part 2 rounds.
	Interference.	Assessment of the difficulty in adapting to the changes.	Number of correct #1 ice creams delivered without looking at the recipe book in Part 2.
	Perseveration.	Measured as the tendency to maintain old habits or strategies that are no longer appropriate.	Number of perseverations when making ice cream in Part 2.
	Switching.	The ability to quickly switch between different information or tasks.	Learning potential in terms of flexibility when making ice cream #4 in Part 2 (which was ice cream #1 in Part 1). Learning potential in terms of flexibility when making ice cream #1 in Part 2 (which is different from ice cream #1 in Part 1).

This multifaceted approach facilitates a meticulous evaluation of executive functions, offering insights into diverse facets of cognitive performance and adaptability.

### 2.4 Procedure

The Nesplora Ice Cream test was administered by evaluators recruited by Giunti Nesplora SL, the company responsible for developing the tool. These evaluators received comprehensive training on using the VR equipment and administering the test to ensure consistency and accuracy in the assessment process. The selected participant was taken to a quiet room to minimize external distractions and ensure a controlled environment. Participants were provided with detailed information about the study, VR assessment, and their rights as participants, including voluntary participation and the ability to withdraw from the study at any time and for any reason. Informed consent was obtained prior to participation. Following informed consent, participants were asked to complete a socio-demographic questionnaire, including information on their age, gender, educational background, occupation, and spoken languages.

Subsequently, participants were introduced to the VR headset and controllers, followed by a session to familiarize them with the system. The Nesplora Ice Cream test includes a thorough usability phase before the assessment phase. This phase simulates the structure and demands of the assessment, helping participants become familiar with the environment and interactions, such as using the headset and controllers, following auditory instructions, and engaging with virtual devices (e.g., ice cream machine, recipe book, trash bin). It also ensures that test outcomes reflect participants’ executive functioning, not just their adaptability to technology. Participants proceed to the assessment test only after successfully completing the usability phase and demonstrating adequate interaction with the VR system.

Throughout the duration of the test (approximately 35 min), the evaluator observed the participant’s progress via monitors that mirrored what was displayed on the participant’s device. This allowed the evaluator to ensure the participant received support in case any issue arose. No incidents were reported during the data collection process: technical problems (software or hardware), difficulties in understanding instructions, fatigue, dizziness, or comprehension challenges.

### 2.5 Data analysis

The statistical analyses and data management were conducted using R version 4.4.1, utilizing several key libraries (R Core Team, 2021). The readr package was employed for reading CSV files (Wickham et al., 2024a), while the tidyr and dplyr packages were used for data manipulation (Wickham et al., 2023; Wickham et al., 2024b). The stringr package was used for string manipulation (Wickham, 2023). The psych package was used for psychometric analysis (William Revelle, 2024), and the MVN package was used for multivariate normality tests (Korkmaz et al., 2014). For data visualization, the ggplot2 package was used to generate graphs (Wickham, 2016), complemented by the ggpubr package for annotations and graphical adjustments (Kassambara, 2023).

First, a description of variables for the total sample was conducted, followed by an examination of sex and age differences within the normative sample. Normative groups were established, and analyses of homoscedasticity and normality were conducted.

Construct validity, as defined by Messick (1980), is emphasized as the overarching framework for test validation, integrating content and criterion validity. Factor analysis was employed to assess factorial validity, with the study noting that convergent-discriminant validity was not addressed due to the interrelated nature of the variables. The study acknowledged the conceptual nature of the assumptions underlying factor analysis, noting that the requirements fo normality and homoscedasticity could be less stringent in this context (Cronbach, 1988).

Multicollinearity was assessed using the determinant of the correlation matrix and Bartlett’s test of sphericity. The Kaiser-Meyer-Olkin (KMO) measure was used to determine sample adequacy for factor analysis (Shrestha, 2021). To enhance the interpretability of factor loadings, promax rotation was used during the factor rotation procedures.

The adaptive nature of the Nesplora Ice Cream test was emphasized, enhancing its ecological validity but complicating the estimation of traditional reliability. Cronbach’s alpha and McDonald’s omega were used to evaluate internal consistency, with values above 0.7 or 0.8 considered sufficient for scale reliability (Ventura-León and Caycho-Rodríguez, 2017).

Difficulty and discrimination indices were calculated as indicators of test quality. The “difficulty index” commonly used to describe the ratio of correct answers to the maximum score, was more accurately referred to as the “ease index” indicating how easy a question was, according to Moreno et al. (2005).

## 3 Results

In this section, the results of the test conducted in Spain on individuals aged 17 to 80 are showed, aimed at obtaining normative data for the Ice Cream Test.

Table 2 presents the distribution of the normative sample by sex and age, while Table 3 provides a detailed description of the variable results for the total sample (*n* = 419).

**Table 2:** Distribution of the sample by age and gender.

Age	Sex	Total	Percentage
17–30	Female	42	10.02
17–30	Male	41	9.79
31–60	Female	138	32.94
31–60	Male	128	30.55
61–80	Female	34	8.11
61–80	Male	36	8.59

The sample size is 419.

**Table 3:** Description of variable results for the total sample (*n* = 419).

	Mean	SD	Q1	Median	Q3	Max	Skew	Kurtosis
Number of shifts correctly assigned in Part 1	4.43	2.35	2	5	7	7	−0.38	−1.27
Number of shifts correctly assigned in Part 2	4.39	2.68	2	6	7	7	−0.43	−1.44
Learning potential to identify whether the customer wears a neoprene suit	148.99	100.22	37	192	242	242	−0.44	−1.54
Learning potential when it comes to assign the right order to the customers	150.60	142.04	0	120	288	341	0.18	−1.71
Number of total correct ice creams delivered correctly without looking at the recipe book on Part 1 rounds	20.26	9.30	16	24	27	28	−1.18	0
Number of correct #1 ice creams delivered without looking at the recipe book in Part 1 rounds.	8.95	4.01	8	11	12	12	−1.26	0.20
Number of correct #1 ice creams delivered without looking at the recipe book in Part 2.	6.39	3.44	4	8	9	10	−0.64	−0.99
Number of correct #1 ice creams delivered without looking at the recipe book in Part 2.	15.52	8.71	8	17	23	28	−0.33	−1.16
Learning potential in relation to making ice cream #1 correctly	95.71	67.89	16	114	164	164	−0.32	−1.58
Learning potential in terms of flexibility when making ice cream #4 in Part 2 (which was ice cream #1 in Part 1)	42.47	53.05	0	9	85.50	147	0.91	−0.71
Number of perseverations when making the ice creams in Part 2	2.48	2.72	0.	2	4	17	1.47	2.68
Learning potential in terms of flexibility when making ice cream #1 in Part 2 (which is different from ice cream #1 in Part 1)	40.60	49.52	0	10	77	125	0.78	−1.05

The sample size is 419 and the minimum for each variable is 0.

Most variables in the sample are asymmetrically distributed, complicating the use of common parametric statistical tests that assume normality. To address this, specific methods that assume non-normal distributions were used instead of data transformations (Brown and Forsythe, 1974). The normality of the sample by sex was tested using a data energy test, which measures distances between data points (Székely and Rizzo, 2017). This method, derived from Newton’s gravitational potential energy, is effective even for complex data and has shown high accuracy in studies on multivariate normality. The concept parallels Einstein’s equation, E = mc^2^, linking energy with observations and data.

To verify normality for each variable considering sex, the non-parametric Anderson-Darling test was used (Marsaglia and Marsaglia, 2004). This test, a modification of the Kolmogorov–Smirnov test (Shapiro et al., 1968), gives more weight to the tails and uses a specific distribution to calculate critical values. This allows for a more sensitive test but requires calculating critical values for each distribution.

An Anderson-Darling Test on the male subset for the selected variables (Supplementary Table 1) showed non-normality with a *p*-value below 0.001 (df = 5.74), and on the female subset for the selected variables (Supplementary Table 2) showed non-normality with a *p*-value below 0.001 (df = 5.13).

Given the asymmetric distribution of many variables, the Brown-Forsythe test is chosen, using the median as its central statistic (Brown and Forsythe, 1974). This test is robust against various types of non-normal data while maintaining good statistical power. It allows for testing the equality of variance across two or more populations, regardless of group size. Supplementary Table 3 presents the homoscedasticity results by sex.

Since homoscedasticity is equal between the groups of men and women in the planning, learning, and flexibility subtests, separating normative groups by sex is unnecessary.

**Figure 2:**
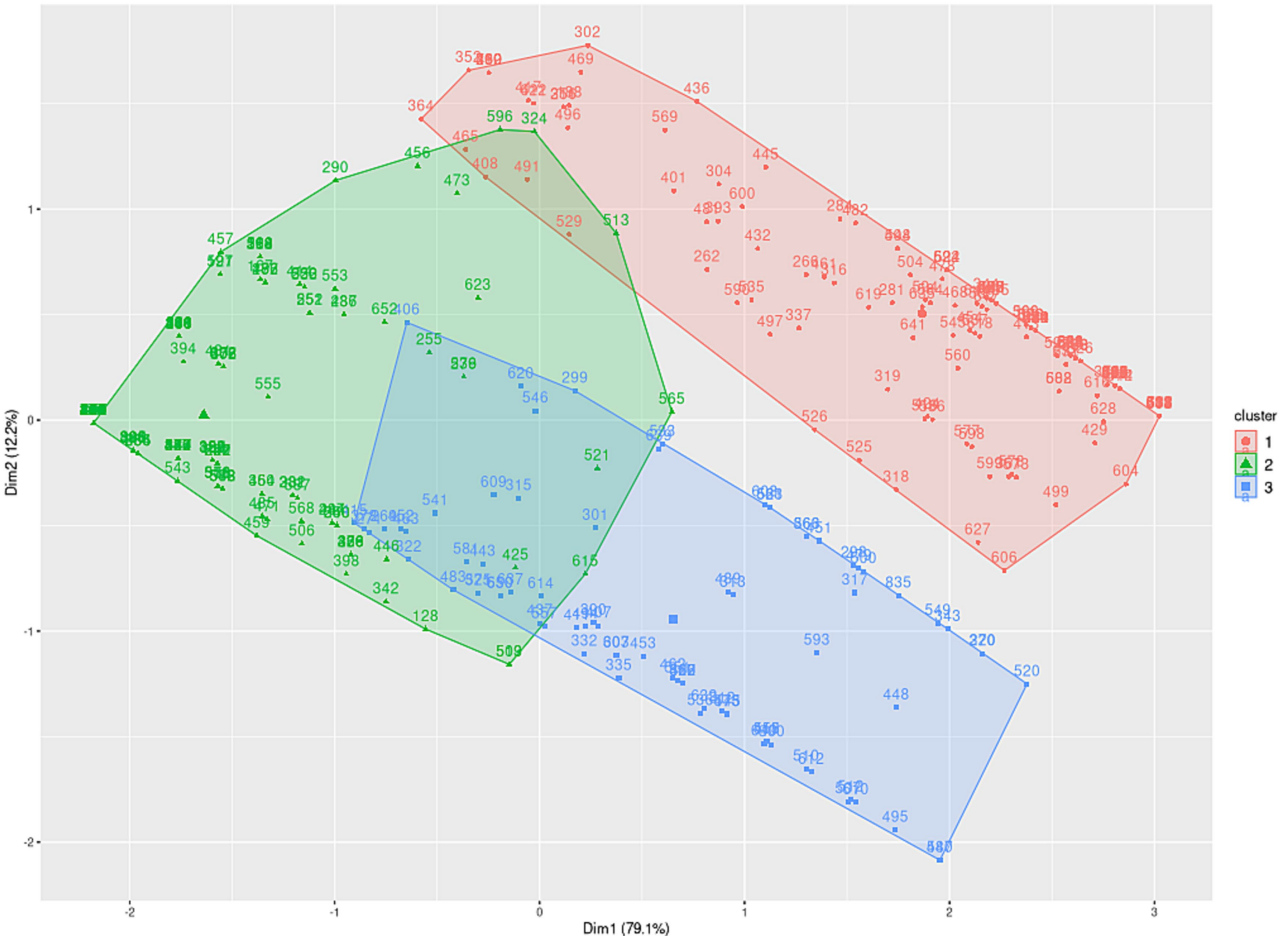
Distribution of planning performance metrics among individuals aged 17 to 80 years.

**Figure 3:**
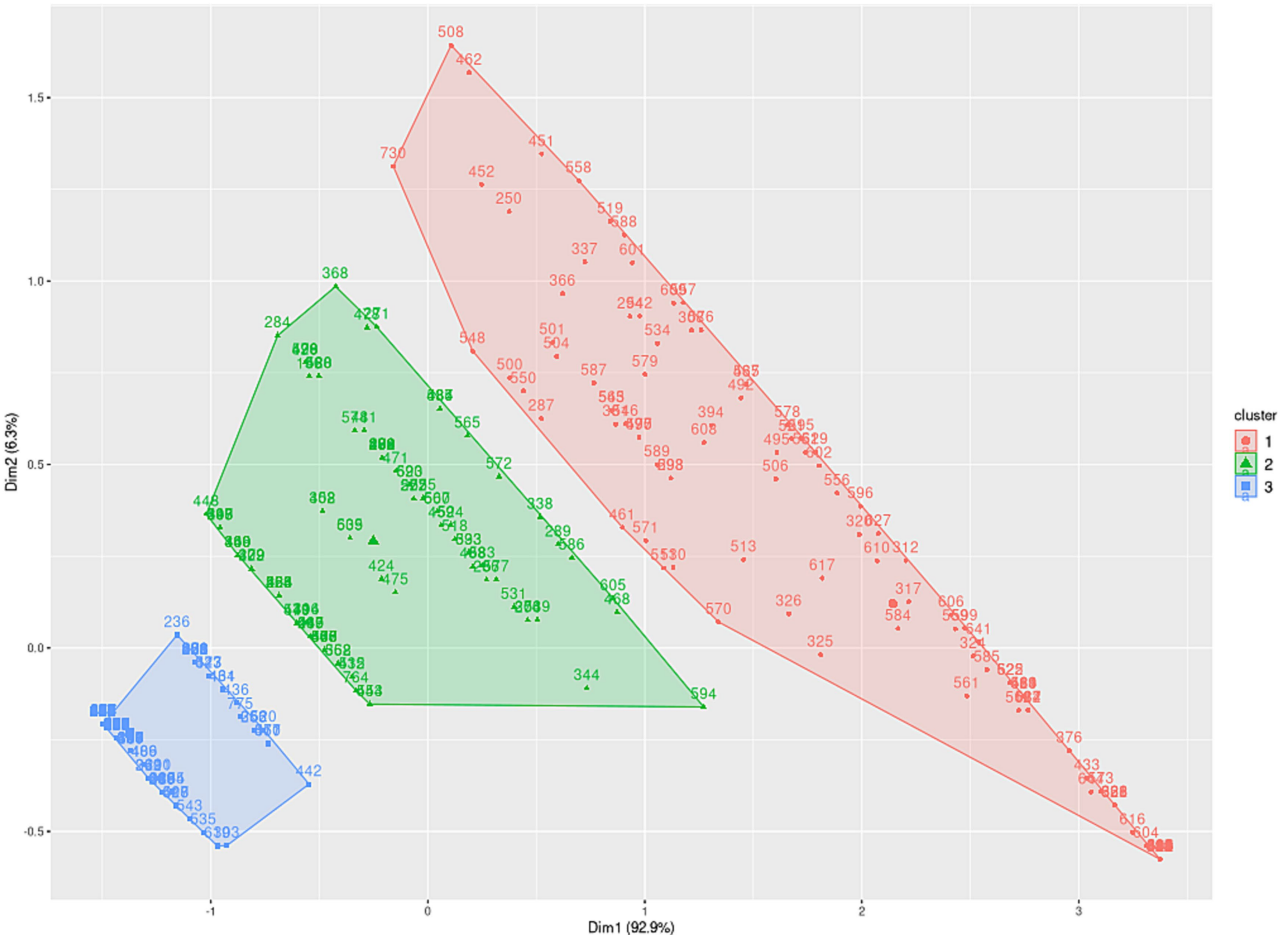
Distribution of learning performance metrics among individuals aged 17 to 80 years.

**Figure 4:**
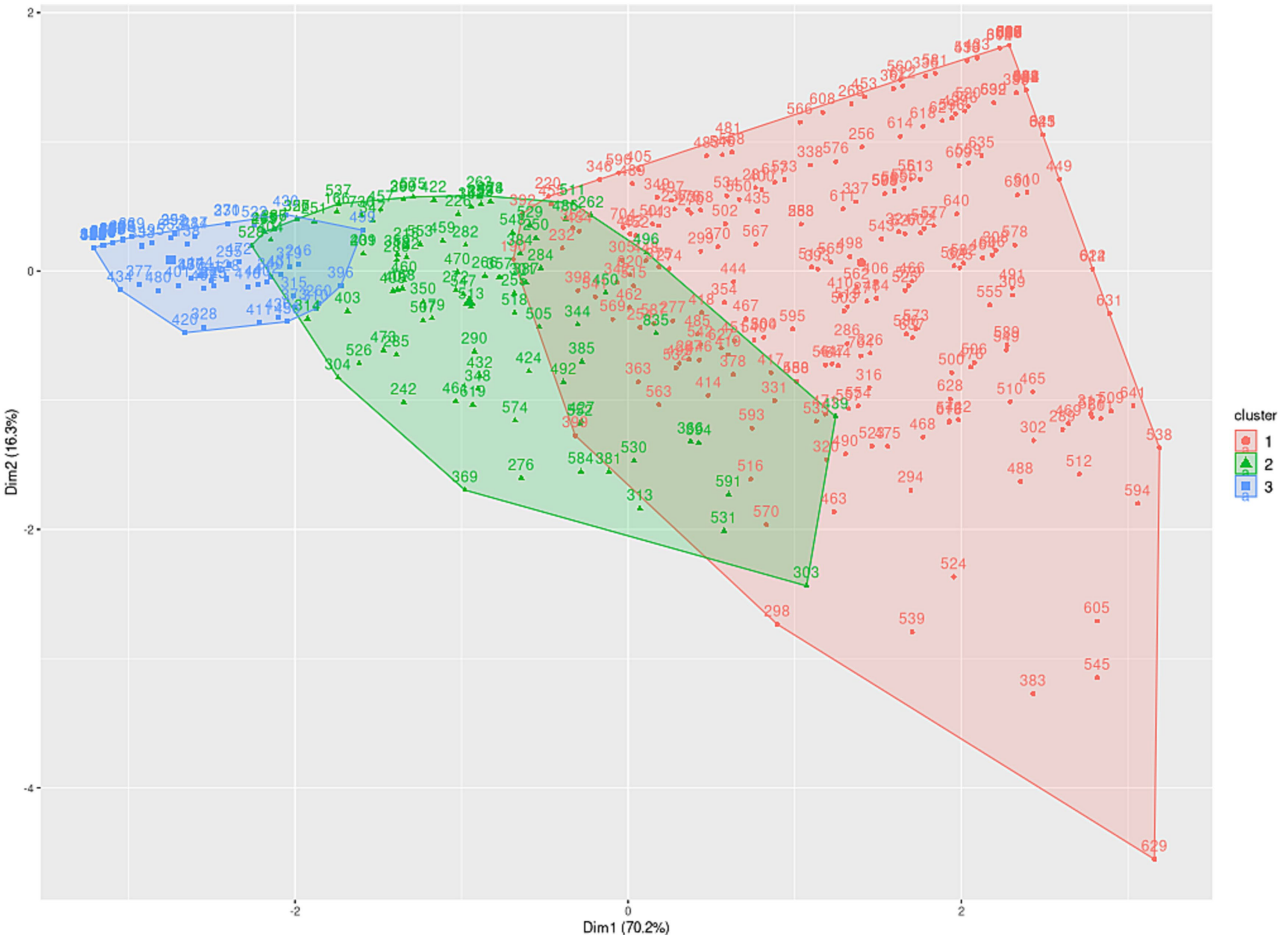
Distribution of flexibility performance metrics among individuals aged 17 to 80 years.

In order to establish age-based scales, three distinct age groups (ages 17 to 80) were identified for the subtests: planning (17–40, 41–61, 62–80), learning (17–44, 45–61, 62–80), and flexibility (17–20, 21–36, 37–80), as illustrated in Figures 2–4, and extensively detailed in Supplementary Tables 4–8. The two principal dimensions represent over 85% of the sample.

Initially, the groups were established based on patterns visually identified within the clusters of variables constituting the psychological constructs of the test (clustering process). Subsequently, these groups underwent validation via hypothesis testing to ascertain the existence of distinct entities within each group.

Given that the subtests do not have shared variables, the normative ranges were restructured without altering the scale, prior to the harmonization of the ranges. This methodology enabled researchers to preserve psychometric precision while concurrently simplifying the test framework, thereby enhancing its interpretability and applicability. The resultant age demographics are delineated as follows: 17–20, 21–36, 37–40, 41–44, 45–61, and 62–80. Table 4 presents the sample distribution among these age brackets.

**Table 4:** Sample distribution by clustered age groups.

Years	Sex	Total	Percentage per age cluster
17–20	Female	7	50
17–20	Male	7	50
21–36	Female	68	51.52
21–36	Male	64	48.48
37–40	Female	21	53.85
37–40	Male	18	46.15
41–44	Female	21	61.76
41–44	Male	13	38.24
45–61	Female	65	48.15
45–61	Male	70	51.85
62–80	Female	32	49.23
62–80	Male	33	50.77

The sample size is 419.

To verify the normality of the normative groups, the same Energy test used to evaluate the normality of the sample by sex is applied. Since the results, which will be detailed later, indicate non-normality, the Brown-Forsythe test is used to determine homoscedasticity between the groups.

### 3.1 Planning

Normality for Planning subtest for the over 17 age scale is shown below. Table 5 shows the data for the 17 to 40 years old Planning cluster. No variables show a normal distribution, and there is heteroscedasticity.

**Table 5:** Planning variable with respect to age 17–40: descriptives and normality tests (Anderson-Darling test) and homoscedasticity test (Brown-Forsythe test) and multivariate normality (E-statistic test).

Variable	Mean	Std.Dev	Median	Max	25th	75th	Skew	Kurtosis	df (A-D test)	BF|denom. df	df (E-test)
Number of shifts correctly assigned in Part 1	5.34	1.97	6	7	4	7	−0.99	−0.18	13.9515*	69.482|337.156*	19.24*
Number of shifts correctly assigned in Part 2	5.50	2.20	7	7	4	7	−1.23	0.04	23.9857*	64.827|319.565*	
Learning potential to identify whether the customer wears a neoprene suit	166.32	94.12	242	242	65	242	−0.73	−1.16	20.3322*	22.955|273.843*	
Learning potential when it comes to assign the right order to the customers	195.38	136.10	236	341	42	341	−0.36	−1.55	12.3531*	43.716|406.658*	

The sample size is 185 and the minimum of each variable is 0. Besides, all ‘num. df’ values in the Brown-Forsythe test are 2. ^*^All variables show NOT normality or NOT homoscedasticity with a *p*-value < 0.001.

Table 6 shows the data for the 41 to 61 years old Planning cluster. No variables show a normal distribution, and there is heteroscedasticity.

**Table 6:** Planning variable with respect to age 41–61: descriptives and normality tests (Anderson-Darling test) and homoscedasticity test (Brown-Forsythe test) and multivariate normality (E-statistic test).

Variable	Mean	Std.Dev	Median	Max	25th	75th	Skew	Kurtosis	df (A-D test)	BF|denom. df	df (E-test)
Number of shifts correctly assigned in Part 1	4.37	2.27	5	7	2	7	−0.32	−1.22	5.9707*	69.482|337.156*	8.02*
Number of shifts correctly assigned in Part 2	4.18	2.64	5	7	2	7	−0.31	−1.50	9.6676*	64.827|319.565*	
Learning potential to identify whether the customer wears a neoprene suit	158.01	97.30	192	242	65	242	−0.60	−1.34	16.4168*	22.955|273.843*	
Learning potential when it comes to assign the right order to the customers	145.27	142.39	91	341	0	288	0.25	−1.69	13.4906*	43.716|406.658*	

The sample size is 169 and the minimum of each variable is 0. Besides, all ‘num. df’ values in the Brown-Forsythe test are 2. ^*^All variables show NOT normality or NOT homoscedasticity with a *p*-value < 0.001.

Table 7 shows the data for the 62 to 80-year-old Planning cluster. No variables show a normal distribution, and there is heteroscedasticity.

**Table 7:** Planning variable with respect to age 62–80: descriptives and normality tests (Anderson-Darling test) and homoscedasticity test (Brown-Forsythe test) and multivariate normality (E-statistic test).

Variable	Mean	Std.Dev	Median	Max	25th	75th	Skew	Kurtosis	df (A-D test)	BF|denom. df	df (E-test)
Number of shifts correctly assigned in Part 1	2	1.70	2	7	1	3	1.08	0.53	53.1555*	69.482|337.156*	4.16*
Number of shifts correctly assigned in Part 2	1.78	2.05	1	7	0	2	1.19	0.45	4.2244*	64.827|319.565*	
Learning potential to identify whether the customer wears a neoprene suit	76.17	93.84	18	242	0	146	0.78	−1.03	6.4716*	22.955|273.843*	
Learning potential when it comes to assign the right order to the customers	37.03	79.44	0	341	0	24	2.41	4.89	12.7472*	43.716|406.658*	

The sample size is 65 and the minimum of each variable is 0. Besides, all ‘num. df’ values in the Brown-Forsythe test are 2. ^*^All variables show NOT normality or NOT homoscedasticity with a *p*-value < 0.001.

### 3.2 Learning

Normality for Learning subtest for the over 17 age scale is shown below. Table 8 shows the data for the 17 to 44 years old Learning cluster. No variables show a normal distribution, and there is heteroscedasticity.

**Table 8:** Learning variable with respect to age 17–44: descriptives and normality tests (Anderson-Darling test) and homoscedasticity test (Brown-Forsythe test) and multivariate normality (E-statistic test).

Variable	Mean	Std.Dev	Median	Max	25th	75th	Skew	Kurtosis	df (A-D test)	BF|denom. df	df (E-test)
Number of total correct ice creams delivered correctly without looking at the recipe book on Part 1 rounds	24.77	5.22	27	28	24	28	−2.67	7.96	24.4882*	132.781|197.539*	40.83*
Number of correct #1 ice creams delivered without looking at the recipe book in Part 1 rounds	10.83	2.24	12	12	10	12	−2.91	9.61	31.4413*	117.207|183.474*	
Learning potential in relation to making ice cream #1 correctly	129.05	52.24	164	164	114	164	−1.28	0.28	28.3040*	129.418|315.618*	

The sample size is 219 and the minimum of each variable is 0. Besides, all ‘num. df’ values in the Brown-Forsythe test are 2. ^*^All variables show NOT normality or NOT homoscedasticity with a *p*-value < 0.001.

Table 9 shows the data for the 45 to 61 years old Learning cluster. No variables show a normal distribution, and there is heteroscedasticity.

**Table 9:** Learning variable with respect to age 45–61: descriptives and normality tests (Anderson-Darling test) and homoscedasticity test (Brown-Forsythe test) and multivariate normality (E-statistic test).

Variable	Mean	Std.Dev	Median	Max	25th	75th	Skew	Kurtosis	df (A-D test)	BF|denom. df	df (E-test)
Number of total correct ice creams delivered correctly without looking at the recipe book on Part 1 rounds	19.39	8.44	22	28	16	26	−1.08	0.10	6.6674*	132.781|197.539*	6.27*
Number of correct #1 ice creams delivered without looking at the recipe book in Part 1 rounds	8.62	3.66	10	12	7.	11	−1.18	0.29	8.3284*	117.207|183.474*	
Learning potential in relation to making ice cream #1 correctly	80.30	63.13	74	164	12.50	138	0.06	−1.53	5.7485*	129.418|315.618*	

The sample size is 135 and the minimum of each variable is 0. Besides, all ‘num. df’ values in the Brown-Forsythe test are 2. ^*^All variables show NOT normality or NOT homoscedasticity with a *p*-value < 0.001.

Table 10 shows the data for the 62 to 80 years old Learning cluster. No variables show a normal distribution, and there is heteroscedasticity.

**Table 10:** Learning variable with respect to age 62–80: descriptives and normality tests (Anderson-Darling test) and homoscedasticity test (Brown-Forsythe test) and multivariate normality (E-statistic test).

Variable	Mean	Std.Dev	Median	Max	25th	75th	Skew	Kurtosis	df (A-D test)	BF|denom. df	df (E-test)
Number of total correct ice creams delivered correctly without looking at the recipe book on Part 1 rounds	6.92	8.43	3	27	0	14	0.92	−0.52	5.1163*	132.781|197.539*	11.71*
Number of correct #1 ice creams delivered without looking at the recipe book in Part 1 rounds	3.31	3.90	2	12	0	7	0.83	−0.76	4.8949*	117.207|183.474*	
Learning potential in relation to making ice cream #1 correctly	15.42	40.16	0	164	0	0	2.74	6.59	16.3*	129.418|315.618*	

The sample size is 65 and the minimum of each variable is 0. Besides, all ‘num. df’ values in the Brown-Forsythe test are 2. ^*^All variables show NOT normality or NOT homoscedasticity with a *p*-value < 0.001.

### 3.3 Flexibility

Finally, normality for Flexibility subtest for the over 17 age scale is shown below. Table 11 shows the data for the 17 to 20-year-old cluster. No variables show a normal distribution, and there is heteroscedasticity.

**Table 11:** Flexibility variable with respect to age 17–20: descriptives and normality tests (Anderson-Darling test) and homoscedasticity test (Brown-Forsythe test) and multivariate normality (E-statistic test).

Variable	Mean	Std.Dev	Median	Max	25th	75th	Skew	Kurtosis	df (A-D test)	BF|denom. df	df (E-test)
Number of total correct ice creams delivered correctly without looking at the recipe book on Part 2 rounds	23.57	2.59	24	18	27	22	25.75	−0.57	0.4661*	88.078|306.731*	0.65*
Number of correct #1 ice creams delivered without looking at the recipe book in Part 2.	9.07	0.92	9	7	10	9	10	−0.68	0.9342*	81.748|351.204*	
Number of perseverations when making the ice creams in Part 2	0.71	0.91	0	0	2	0	1.75	0.53	1.8494*	34.986|305.039*	
Learning potential in terms of flexibility when making ice cream #4 in Part 2 (which was ice cream #1 in Part 1)	80.93	30.31	85.50	20	147	59	97	0.07	0.6693*	39.913|148.425*	
Learning potential in terms of flexibility when making ice cream #1 in Part 2 (which is different from ice cream #1 in Part 1)	74.29	38.87	77	10	125.00	47.50	11	0.07	0.5596*	23.881|84.084*	

The sample size is 14 and the minimum of each variable is 0. Besides, all ‘num. df’ values in the Brown-Forsythe test are 2. ^*^All variables show NOT normality or NOT homoscedasticity with a *p*-value < 0.001.

Table 12 shows the data for the 21-to-36-year-old Planning cluster. No variables show a normal distribution, and there is heteroscedasticity.

**Table 12:** Flexibility variable with respect to age 21–36: descriptives and normality tests (Anderson-Darling test) and homoscedasticity test (Brown-Forsythe test) and multivariate normality (E-statistic test).

Variable	Mean	Std.Dev	Median	Max	25th	75th	Skew	Kurtosis	df (A-D test)	BF|denom. df	df (E-test)
Number of total correct ice creams delivered correctly without looking at the recipe book on Part 2 rounds	20.12	6.75	22	28	17	26	−0.92	−0.14	4.7535*	88.078|306.731*	7.05*
Number of correct #1 ice creams delivered without looking at the recipe book in Part 2.	8.11	2.36	9	10	7	10	−1.52	1.63	10.0984*	81.748|351.204*	
Number of perseverations when making the ice creams in Part 2	1.58	1.96	1	9	0	2	1.45	1.70	9.4834*	34.986|305.039*	
Learning potential in terms of flexibility when making ice cream #4 in Part 2 (which was ice cream #1 in Part 1)	67.20	57.10	54	147	9	121	0.19	−1.53	6.2709*	39.913|148.425*	
Learning potential in terms of flexibility when making ice cream #1 in Part 2 (which is different from ice cream #1 in Part 1)	59.58	51.88	58	125	4	125	0.14	−1.67	8.7413*	23.881|84.084*	

The sample size is 132 and the minimum of each variable is 0. Besides, all ‘num. df’ values in the Brown-Forsythe test are 2. ^*^All variables show NOT normality or NOT homoscedasticity with a *p*-value < 0.001.

Table 13 shows the data for the 37 to 80 years old Planning cluster. No variables show a normal distribution, and there is heteroscedasticity.

**Table 13:** Flexibility variable with respect to age 37–80: descriptives and normality tests (Anderson-Darling test) and homoscedasticity test (Brown-Forsythe test) and multivariate normality (E-statistic test).

Variable	Mean	Std.Dev	Median	Max	25th	75th	Skew	Kurtosis	df (A-D test)	BF|denom. df	df (E-test)
Number of total correct ice creams delivered correctly without looking at the recipe book on Part 2 rounds	12.89	8.59	13	28	6	20	0.03	−1.23	3.6831*	88.078|306.731*	10.48*
Number of correct #1 ice creams delivered without looking at the recipe book in Part 2.	5.43	3.57	6	10	2	9	−0.22	−1.39	8.5347*	81.748|351.204*	
Number of perseverations when making the ice creams in Part 2	3.01	2.94	2	17	1	5	1.27	1.95	9.4536*	34.986|305.039*	
Learning potential in terms of flexibility when making ice cream #4 in Part 2 (which was ice cream #1 in Part 1)	28.54	46.29	0	147	0	36	1.52	0.88	40.3061*	39.913|148.425*	
Learning potential in terms of flexibility when making ice cream #1 in Part 2 (which is different from ice cream #1 in Part 1)	29.69	45.24	0	125	0	58	1.27	0.01	39.7000*	23.881|84.084*	

The sample size is 273 and the minimum of each variable is 0. Besides, all ‘num. df’ values in the Brown-Forsythe test are 2. ^*^All variables show NOT normality or NOT homoscedasticity with a *p*-value < 0.001.

Given the differences in variances between the groups, it is justified to create separate normative scales for each group. This is because the differences in variance reflect distinctive characteristics of the groups that influence data dispersion (Harris and Boyd, 1990).

**Figure 5:**
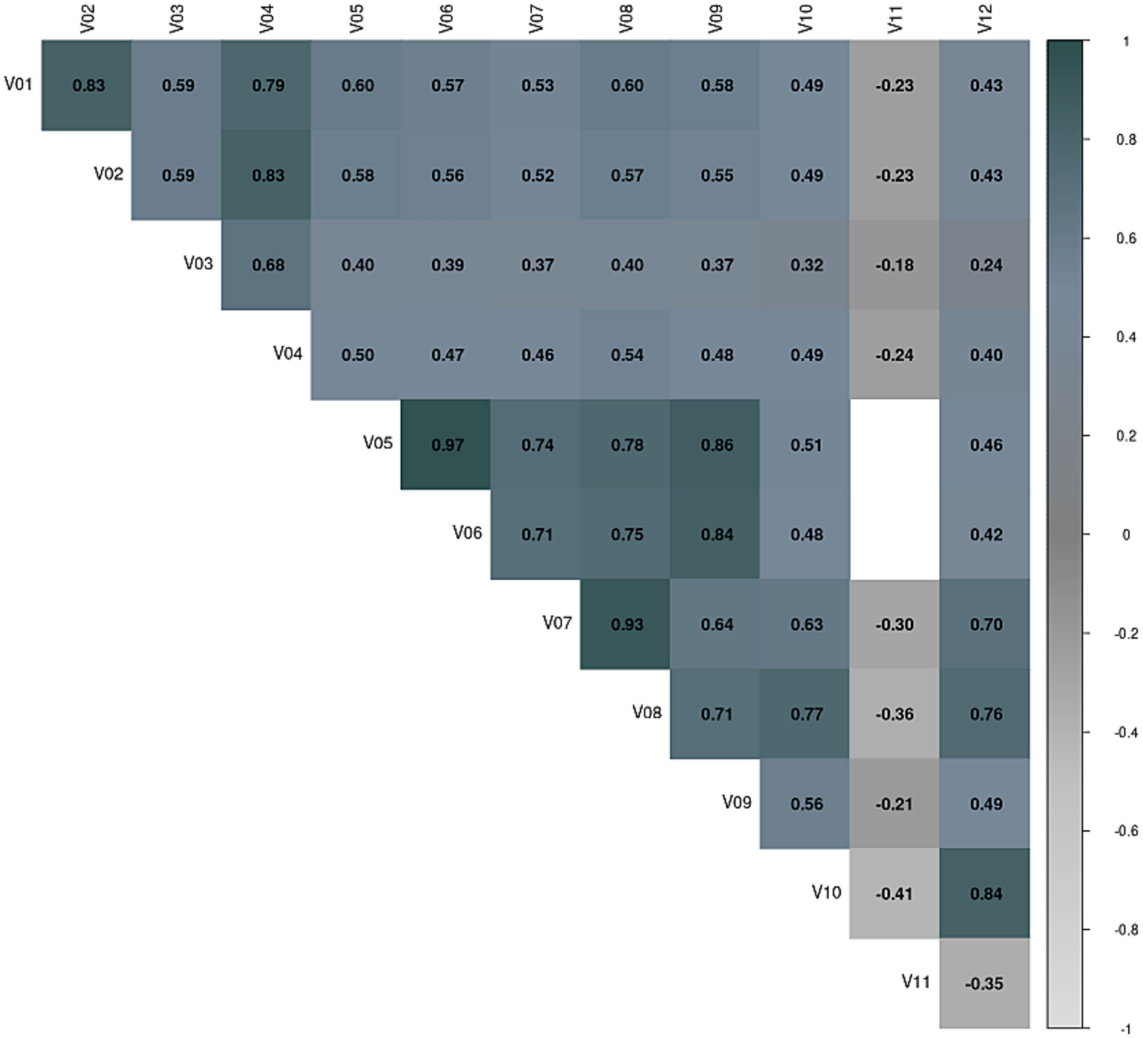
Ice Cream VR test. Variable correlation matrix. V01: Number of shifts correctly assigned in Part 1. V02: Number of shifts correctly assigned in Part 2. V03: Learning potential to identify whether the customer wears a neoprene suit or not, (measured at Round 13). V04: Learning potential when it comes to assign the right order to the customers. V05: Number of total correct ice creams delivered correctly without looking at the recipe book on Part 1 rounds. V06: Number of correct #1 ice creams delivered without looking at the recipe book in Part 1 rounds. V07: Number of correct #1 ice creams delivered without looking at the recipe book in Part 2. V08: Number of total correct ice creams delivered correctly without looking at the recipe book on Part 2 rounds. V09: Learning potential in relation to making ice cream #1 correctly. V10: Learning potential in terms of flexibility when making ice cream #4 in Part 2 (which was ice cream #1 in Part 1). V11: Number of perseverations when making the ice creams in Part 2. V12: Learning potential in terms of flexibility when making ice cream #1 in Part 2 (which is different from ice cream #1 in Part 1).

Visual inspection revealed a significant number of correlations above. 0.3, justifying the conduction of the factor analysis (Figure 5).

The result (Barlett Statistic = 1318.92, df = 66, *p* < 0.0001) implied the existence of correlated variables and, therefore, indicate a factor analysis can be applied and the KMO, as shown in Supplementary Table 9, all values obtained were higher than 0.75 (KMO = 0.86).

Therefore, it is acceptable to perform a factor analysis. The results of the factor analysis were as shown below in Table 14.

**Table 14:** Factor analysis results.

Variable	Planning	Learning	Flexibility
Number of shifts correctly assigned in Part 1	**0.816**	0.101	0.02
Number of shifts correctly assigned in Part 2	**0.899**	0.06	−0.029
Learning potential to identify whether the customer wears a neoprene suit	**0.702**	0.006	−0.036
Learning potential when it comes to assign the right order to the customers	**0.954**	−0.075	0.001
Number of total correct ice creams delivered correctly without looking at the recipe book on Part 1 rounds	0.03	**0.93**	0.08
Number of correct #1 ice creams delivered without looking at the recipe book in Part 1 rounds.	0.015	**0.944**	0.046
Number of correct #1 ice creams delivered without looking at the recipe book in Part 2.	−0.1	0.302	**0.786**
Number of total correct ice creams delivered correctly without looking at the recipe book on Part 2 rounds	−0.043	0.273	**0.843**
Learning potential in relation to making ice cream #1 correctly	0.094	**0.73**	0.124
Learning potential in terms of flexibility when making ice cream #4 in Part 2 (which was ice cream #1 in Part 1)	0.112	−0.027	**0.744**
Number of perseverations when making the ice creams in Part 2	−0.109	0.261	**−0.501**
Learning potential in terms of flexibility when making ice cream #1 in Part 2 (which is different from ice cream #1 in Part 1)	0	−0.065	**0.828**

Highest factorial loading for each variable is in bold. The sign indicates direction.

The Factorial Analysis conducted accounts for 69.3% of the variance. The remaining unexplained variance by the three factors (‘planning’, ‘learning’, ‘flexibility’) is detailed in Supplementary Table 10.

To conclude, Supplementary Tables 11–13 provide data on test reliability and internal consistency.

## 4 Discussion

The present study confirms the relevance of Nesplora Ice Cream test as an innovative virtual reality (VR)-based neuropsychological assessment designed to evaluate EF in context with high ecological validity. This research not only meets the initial objective of establishing normative data for adults aged 17–80 years but also offers a deeper understanding of the factor structure or psychological construct of executive functions in this population. These findings underscore the potential of virtual reality technology to bridge the gap between traditional neuropsychological assessments and real-world scenarios, providing a more accurate and personalized evaluation of cognitive processes. The ecological validity of this approach is further enhanced by combining virtual reality with a well-constructed dynamic design based on tasks requiring planning, learning, and flexibility.

Regarding the primary objectives, the results highlight three key factors of executive functions: planning, learning, and cognitive flexibility, which explain more than 69% of the total variance. Each factor (psychological construct) is composed of specific variables that allow for a precise assessment of these skills. The use of cluster analysis revealed differentiated age groups for each function, illustrating the developmental trajectory of executive functions throughout adulthood. Specifically, planning showed different age groups of 17–40, 41–61 and 62–80 years; learning was divided into ranges 17–44, 45–61 and 62–80 years; and cognitive flexibility exhibited a division into groups 17–20, 21–36, and 37–80 years. It is important to note that the executive functions are complex constructs made up of various underlying variables. This comprehensive approach captures the nuanced nature of their development in adulthood, offering a holistic understanding of cognitive development beyond what individual measures can provide.

The results of this study align with the existing literature on changes in executive function (EF) across the lifespan, supporting theoretical models describing the evolution of executive functions with age (Diamond, 2013; Luna et al., 2004; Park and Reuter-Lorenz, 2009; Salthouse, 2010). In addition, these findings are consistent with previous studies that highlight VR’s ability to facilitate the creation of controlled environments that better reflect real-life challenges, allowing for a more accurate and individualized assessment (Adriasola et al., 2024; Cañada et al., 2024; Pieri et al., 2023). As mentioned in the introduction, the integration of VR into EF assessment has been extensively explored in recent literature, with pioneering studies demonstrating its ecological advantages over conventional methods. For example, Bohil et al. (2011) highlighted how VR environments allow precise control over experimental conditions while simulating real-world scenarios, bridging the gap between laboratory settings and everyday cognitive demands. This foundational research informed the development of validated tools such as the Virtual Multiple Errands Test (VMET; Cipresso et al., 2014) and the Virtual Environment Grocery Store (VEGS; Parsons and McMahan, 2017), which strongly correlate with traditional neuropsychological tests, such as the Trail Making Test (TMT; Raspelli et al., 2012).

Recent advancements underscore the clinical utility of VR in detecting EF deficits that might be missed by traditional methods. For example, studies utilizing VEGS have revealed a decrease in multitasking abilities among older adults under high-distraction conditions, a finding undetectable by paper-and-pencil assessments (Nir-Hadad et al., 2023; Kizony et al., 2017). A systematic review by Borgnis et al. (2022) analyzed 301 studies and identified 100 VR-based tools validated for evaluating EF subcomponents, including working memory, inhibition, and cognitive flexibility, particularly in clinical populations such as stroke survivors and older adults with mild cognitive impairment. Meta-analytic evidence further supports the concurrent validity of VR evaluations, with pooled correlations against traditional measures (Borgnis et al., 2022).

Furthermore, the comprehensive approach to test validation and data analyses supports the Nesplora Ice Cream test as a robust tool for assessing executive functions in adults, with potential applications in clinical and research settings. Indeed, the psychometric analyses, including factor analysis, multicollinearity checks, and reliability estimations, further strengthened the tool’s validity and reliability. The use of modern validation techniques and the integration of VR technology contribute to a more precise and individualized assessment, offering a significant advancement in neuropsychological testing. Moreover, the lack of significant gender differences in the findings simplifies the interpretation of normative results, further supporting the Nesplora Ice Cream test’s applicability in mixed-gender populations. This reinforces the test’s value as a tool for assessing EF in diverse groups, including clinical populations.

However, the study presents some limitations that should be considered. The external validity of the findings is somewhat restricted, as the normative data are based solely on the Spanish population. This highlights the importance of cross-cultural validation to ensure that the tool can be effectively applied in other cultural and linguistic contexts. Moreover, the convergent validity of the instrument was not fully established, as it was not tested against a broad range of established measures, which limits the ability to confirm the extent to which it correlates with other relevant constructs.

Further studies should aim to address these limitations by expanding the sample to include diverse populations from different cultural, and linguistic backgrounds and by incorporating explicit measures of VR experience to better understand its role in cognitive and behavioral outcomes. This would help assess the external validity of the tool and its applicability in a broader range of settings. Additionally, future research should focus on evaluating the convergent validity of the instrument by comparing it with a wider array of established measures. This would provide a clearer understanding of how well the tool correlates with other relevant constructs and contribute to confirming its overall effectiveness and robustness in measuring the intended variables across different contexts. Educational factors will be introduced in future studies, with an increased sample size to better account for the potential impact of educational background on performance, as previous research suggests that cognitive skills associated with education can influence virtual task engagement (Iriarte et al., 2016). This will allow for a more comprehensive understanding of how educational variables also interact with VR-based assessments and their potential effects on the outcomes measured.

Finally, in terms of practical applications, the Nesplora Ice Cream test shows considerable promise in clinical, educational, and occupational settings. It can identify early signs of EF, facilitating the development of personalized intervention plans. Longitudinal studies could further investigate how aging impacts the executive functions assessed by the Nesplora Ice Cream test. Additionally, exploring its potential in clinical populations, such as individuals with autism spectrum disorders or ADHD, could provide insights into the diagnostic sensitivity of the tool. Integrating complementary technologies, such as artificial intelligence, could enhance the analysis of responses and enable the generation of more detailed and individualized cognitive profiles.

Previous research has been showed the potential of VR in the assessment of EF in different clinical population. For instance, in the context of traumatic brain injuries (TBI), tools like the Virtual Environment Grocery Store (VEGS) assess executive dysfunction through multitasking tasks, predicting outcomes in occupational rehabilitation (Parsons and McMahan, 2017). For neurodegenerative disorders, the Virtual Multiple Errands Test (VMET) can differentiate Parkinson’s disease patients from healthy elder people (Cipresso et al., 2014).

In conclusion, this study marks a significant advance in the use of virtual reality as a neuropsychological tool, demonstrating the potential for innovative approaches in the assessment of cognitive functions. Despite challenges related to generalization of the results and the need for validation in clinical populations, the findings strongly support the efficacy of Nesplora Ice Cream test in assessing key executive functions, including planning, learning, and cognitive flexibility.

These functions are critical to daily life and are often impaired in a range of neuropsychological conditions, making the tool particularly valuable. The use of virtual reality enhances the ecological validity of assessments, offering a comprehensive, dynamic evaluation closer to real-world scenarios than traditional neuropsychological tests. By addressing limitations of paper-based tools, such as low interactivity, the Nesplora Ice Cream test tracks cognitive processes in real time and adapts assessments to individual responses for greater precision.

Beyond research, the tool has significant applications in clinical, educational, and occupational settings, enabling early detection of executive function deficits and supporting personalized interventions. Its adaptability makes it ideal for cognitive rehabilitation and tailored training programs, thereby improving outcomes in diverse populations.

Looking ahead, future studies should validate its use across cultures, age groups, and clinical populations, and explore integration with technologies like artificial intelligence for detailed cognitive profiling. Despite current challenges, the Nesplora Ice Cream test has the potential to transform neuropsychological assessment and rehabilitation, advancing personalized care and expanding understanding of executive functions.

## Data Availability

The raw data supporting the conclusions of this article will be made available by the authors, without undue reservation.
