# Supplementary material for: Nesplora Ice Cream test: a normative study of a virtual reality-based executive function assessment in adults
Source: Front Psychol. 2025 Mar 31;16:1561802. doi: 10.3389/fpsyg.2025.1561802 (PMC11995457; doi:10.3389/fpsyg.2025.1561802)
Supplement: Supplementary file 1 [file Table_1.docx]

Supplementary Material

# Supplementary Tables

Supplementary Table 1. Descriptive data for each variable with respect to sex: male.

| **Variable** | **Mean** | **Std.Dev** | **Median** | **Max** | **25th** | **75th** | **Skew** | **Kurtosis** |
| --- | --- | --- | --- | --- | --- | --- | --- | --- |
| Number of shifts correctly assigned in Part 1 | 4.49 | 2.39 | 5 | 7 | 2 | 7 | -.41 | -1.27 |
| Number of shifts correctly assigned in Part 2 | 4.36 | 2.71 | 5 | 7 | 2 | 7 | -.45 | -1.42 |
| Learning potential to identify whether the customer wears a neoprene suit | 151.02 | 101.81 | 192 | 242 | 37 | 242 | -.48 | -1.54 |
| Learning potential when it comes to assign the right order to the customers | 156.04 | 144.45 | 153 | 341 | 0 | 288 | .11 | -1.76 |
| Number of total correct ice creams delivered correctly without looking at the recipe book on Part 1 rounds | 20.28 | 9.60 | 24 | 28 | 17 | 28 | -1.17 | -.04 |
| Number of correct #1 ice creams delivered without looking at the recipe book in Part 1 rounds. | 8.94 | 4.17 | 11 | 12 | 8 | 12 | -1.29 | .19 |
| Number of correct #1 ice creams delivered without looking at the recipe book in Part 2. | 94.84 | 69.07 | 114 | 164 | 16 | 164 | -.29 | -1.65 |
| Number of correct #1 ice creams delivered without looking at the recipe book in Part 2. | 15.85 | 8.80 | 17 | 28 | 8 | 23 | -.46 | -1.09 |
| Learning potential in relation to making ice cream #1 correctly | 6.62 | 3.43 | 8 | 10 | 4 | 9 | -.82 | -.72 |
| Learning potential in terms of flexibility when making ice cream #4 in Part 2 (which was ice cream #1 in Part 1) | 2.35 | 2.52 | 2 | 13 | 0 | 4 | 1.39 | 1.80 |
| Number of perseverations when making the ice creams in Part 2 | 42.41 | 52.89 | 9 | 147 | 0 | 97 | .90 | -.74 |
| Learning potential in terms of flexibility when making ice cream #1 in Part 2 (which is different from ice cream #1 in Part 1) | 41.66 | 48.78 | 10 | 125 | 0 | 77 | .72 | -1.09 |

*Note.* The sample size is 205 and the minimum for each variable is 0.

Supplementary Table 2. Descriptive data for each variable with respect to sex: female.

| **Variable** | **Mean** | **Std.Dev** | **Median** | **Max** | **25th** | **75th** | **Skew** | **Kurtosis** |
| --- | --- | --- | --- | --- | --- | --- | --- | --- |
| Number of shifts correctly assigned in Part 1 | 4.37 | 2.31 | 5 | 7 | 2.25 | 7 | -.35 | -1.29 |
| Number of shifts correctly assigned in Part 2 | 4.42 | 2.66 | 6 | 7 | 2 | 7 | -.41 | -1.49 |
| Learning potential to identify whether the customer wears a neoprene suit | 147.04 | 98.86 | 169 | 242 | 37 | 242 | -.39 | -1.54 |
| Learning potential when it comes to assign the right order to the customers | 145.40 | 139.82 | 91 | 341 | 0 | 288 | .25 | -1.67 |
| Number of total correct ice creams delivered correctly without looking at the recipe book on Part 1 rounds | 20.25 | 9.03 | 24 | 28 | 16 | 27 | -1.17 | -.01 |
| Number of correct #1 ice creams delivered without looking at the recipe book in Part 1 rounds. | 8.96 | 3.86 | 11 | 12 | 7 | 12 | -1.21 | .12 |
| Number of correct #1 ice creams delivered without looking at the recipe book in Part 2. | 96.55 | 66.89 | 114 | 164 | 27 | 164 | -.36 | -1.52 |
| Number of correct #1 ice creams delivered without looking at the recipe book in Part 2. | 15.21 | 8.64 | 16 | 28 | 8 | 23 | -.20 | -1.23 |
| Learning potential in relation to making ice cream #1 correctly | 6.17 | 3.45 | 7 | 10 | 3 | 9 | -.47 | -1.20 |
| Learning potential in terms of flexibility when making ice cream #4 in Part 2 (which was ice cream #1 in Part 1) | 2.61 | 2.89 | 2 | 17 | 0 | 4 | 1.48 | 2.88 |
| Number of perseverations when making the ice creams in Part 2 | 42.52 | 53.33 | 9 | 147 | 0 | 74 | .91 | -.70 |
| Learning potential in terms of flexibility when making ice cream #1 in Part 2 (which is different from ice cream #1 in Part 1) | 39.58 | 50.30 | 4 | 125 |  | 77 | .83 | -1.03 |

*Note.* The sample size is 214 and the minimum for each variable is 0.

Supplementary Table 3. Homoscedasticity with respect to sex.

| **Variable** | **Brown-Forsythe Statistic** | **denom df** | **p.value** |
| --- | --- | --- | --- |
| Number of shifts correctly assigned in Part 1 | .246 | 414.415 | .62 |
| Number of shifts correctly assigned in Part 2 | .06 | 415.457 | .806 |
| Learning potential to identify whether the customer wears a neoprene suit | .165 | 414.825 | .685 |
| Learning potential when it comes to assign the right order to the customers | .586 | 414.628 | .444 |
| Number of total correct ice creams delivered correctly without looking at the recipe book on Part 1 rounds | .001 | 412.486 | .969 |
| Number of correct #1 ice creams delivered without looking at the recipe book in Part 1 rounds. | .002 | 410.979 | .967 |
| Learning potential in relation to making ice cream #1 correctly | .066 | 414.663 | .798 |
| Number of total correct ice creams delivered correctly without looking at the recipe book on Part 2 rounds | .553 | 415.454 | .458 |
| Number of correct #1 ice creams delivered without looking at the recipe book in Part 2 | 1.804 | 416.467 | .18 |
| Number of perseverations when making the ice creams in Part 2 | 1.007 | 413.395 | 0.316 |
| Learning potential in terms of flexibility when making ice cream #4 in Part 2 (which was ice cream #1 in Part 1) | 0 | 416.493 | .984 |
| Learning potential in terms of flexibility when making ice cream #1 in Part 2 (which is different from ice cream #1 in Part 1) | .185 | 416.935 | .667 |

*Note.* All variables have a ‘num df’ = 1.

Supplementary Table 4. Clustering with respect to age (>16) and subtest: Planning, Learning and Cognitive Flexibility (I).

| **Scale** | **Age** | | | | | | | | | | | | | | |
| --- | --- | --- | --- | --- | --- | --- | --- | --- | --- | --- | --- | --- | --- | --- | --- |
| Planning | 17 | 18 | 19 | 20 | 21 | 22 | 23 | 24 | 25 | 26 | 27 | 28 | 29 | 30 | 31 |
|  | 0 | 1 | 1 | 1 | 1 | 2 | 1 | 1 | 4 | 0 | 3 | 2 | 2 | 3 | 2 |
|  | **3** | **0** | **4** | **4** | **3** | **2** | **5** | **1** | **8** | **0** | **6** | **6** | **5** | **5** | **6** |
|  | 0 | 0 | 0 | 0 | 0 | 1 | 0 | 1 | 1 | 2 | 0 | 1 | 1 | 2 | 1 |
| Learning | 17 | 18 | 19 | 20 | 21 | 22 | 23 | 24 | 25 | 26 | 27 | 28 | 29 | 30 | 31 |
|  | 0 | 0 | 0 | 0 | 1 | 1 | 0 | 0 | 0 | 1 | 0 | 0 | 0 | 1 | 0 |
|  | 0 | 0 | 1 | 1 | 1 | 1 | 0 | 0 | 3 | 0 | 1 | 1 | 3 | 3 | 3 |
|  | **3** | **1** | **4** | **4** | **2** | **3** | **6** | **3** | **10** | **1** | **8** | **8** | **5** | **6** | **6** |
| Cognitive  Flexibility | 17 | 18 | 19 | 20 | 21 | 22 | 23 | 24 | 25 | 26 | 27 | 28 | 29 | 30 | 31 |
|  | 0 | 0 | 1 | 0 | **1** | **2** | **0** | **0** | **3** | **2** | **5** | **1** | **1** | **6** | **3** |
|  | **2** | **1** | **3** | **4** | **2** | **1** | **3** | **2** | **5** | **0** | **0** | **2** | **2** | **1** | **5** |
|  | 1 | 0 | 1 | 1 | **1** | **2** | **3** | **1** | **5** | **0** | **4** | **6** | **5** | **3** | **1** |

Supplementary Table 5. Clustering with respect to age (>16) and subtest: Planning, Learning and Cognitive Flexibility (II).

| **Scale** | **Age** | | | | | | | | | | | | | | |
| --- | --- | --- | --- | --- | --- | --- | --- | --- | --- | --- | --- | --- | --- | --- | --- |
| Planning | 32 | 33 | 34 | 35 | 36 | 37 | 38 | 39 | 40 | 41 | 42 | 43 | 44 | 45 | 46 |
|  | 3 | 1 | 1 | 5 | 7 | 2 | 3 | 2 | 2 | **3** | **2** | **3** | **6** | **1** | **4** |
|  | **8** | **3** | **5** | **3** | **13** | **4** | **2** | **7** | **11** | **2** | **4** | **6** | **5** | **5** | **3** |
|  | 0 | 1 | 1 | 1 | 2 | 1 | 0 | 4 | 1 | **0** | **1** | **0** | **2** | **2** | **4** |
| Learning | 32 | 33 | 34 | 35 | 36 | 37 | 38 | 39 | 40 | 41 | 42 | 43 | 44 | 45 | 46 |
|  | 2 | 1 | 2 | 2 | 2 | 2 | 1 | 2 | 3 | 0 | 2 | 1 | 1 | **3** | **4** |
|  | 4 | 1 | 0 | 1 | 4 | 0 | 2 | 5 | 2 | 2 | 2 | 2 | 5 | **3** | **2** |
|  | **5** | **3** | **5** | **6** | **16** | **5** | **2** | **6** | **9** | **3** | **3** | **6** | **7** | **2** | **5** |
| Cognitive  Flexibility | 32 | 33 | 34 | 35 | 36 | 37 | 38 | 39 | 40 | 41 | 42 | 43 | 44 | 45 | 46 |
|  | **7** | **2** | **2** | **5** | **8** | **5** | **4** | **6** | **5** | **2** | **4** | **3** | **4** | **6** | **5** |
|  | **2** | **2** | **3** | **3** | **3** | 1 | 0 | 2 | 4 | 2 | 1 | 2 | 5 | 1 | 5 |
|  | **2** | **1** | **2** | **1** | **11** | 1 | 1 | 5 | 5 | 1 | 2 | 4 | 4 | 1 | 1 |

Supplementary Table 6. Clustering with respect to age (>16) and subtest: Planning, Learning and Cognitive Flexibility (III).

| **Scale** | **Age** | | | | | | | | | | | | | | |
| --- | --- | --- | --- | --- | --- | --- | --- | --- | --- | --- | --- | --- | --- | --- | --- |
| Planning | 47 | 48 | 49 | 50 | 51 | 52 | 53 | 54 | 55 | 56 | 57 | 58 | 59 | 60 | 61 |
|  | **1** | **3** | **1** | **3** | **1** | **2** | **5** | **4** | **2** | **0** | **2** | **0** | **4** | **2** | **1** |
|  | **2** | **3** | **6** | **9** | **3** | **2** | **4** | **3** | **6** | **2** | **1** | **1** | **1** | **6** | **1** |
|  | **2** | **5** | **1** | **2** | **1** | **3** | **1** | **2** | **2** | **3** | **1** | **4** | **3** | **2** | **3** |
| Learning | 47 | 48 | 49 | 50 | 51 | 52 | 53 | 54 | 55 | 56 | 57 | 58 | 59 | 60 | 61 |
|  | **3** | **4** | **3** | **3** | **2** | **2** | **5** | **6** | **1** | **1** | **3** | **2** | **3** | **4** | **2** |
|  | **2** | **4** | **3** | **2** | **1** | **2** | **3** | **2** | **5** | **2** | **1** | **2** | **4** | **4** | **1** |
|  | **0** | **3** | **2** | **9** | **2** | **3** | **2** | **1** | **4** | **2** | **0** | **1** | **1** | **2** | **2** |
| Cognitive  Flexibility | 47 | 48 | 49 | 50 | 51 | 52 | 53 | 54 | 55 | 56 | 57 | 58 | 59 | 60 | 61 |
|  | **2** | **8** | **6** | **7** | **4** | **5** | **6** | **7** | **9** | **4** | **2** | **4** | **7** | **8** | **3** |
|  | 2 | 3 | 1 | 4 | 0 | 1 | 3 | 2 | 0 | 1 | 2 | 1 | 1 | 1 | 2 |
|  | 1 | 0 | 1 | 3 | 1 | 1 | 1 | 0 | 1 | 0 | 0 | 0 | 0 | 1 | 0 |

Supplementary Table 7. Clustering with respect to age (>16) and subtest: Planning, Learning and Cognitive Flexibility (IV).

| **Scale** | **Age** | | | | | | | | | | | | | | |
| --- | --- | --- | --- | --- | --- | --- | --- | --- | --- | --- | --- | --- | --- | --- | --- |
| Planning | 62 | 64 | 65 | 66 | 67 | 68 | 69 | 70 | 71 | 72 | 73 | 74 | 75 | 76 | 77 |
|  | **3** | **4** | **4** | **0** | **4** | **2** | **4** | **1** | **2** | **3** | **2** | **3** | **5** | **3** | **1** |
|  | 0 | 1 | 0 | 1 | 1 | 1 | 0 | 0 | 1 | 1 | 0 | 0 | 0 | 0 | 0 |
|  | **1** | **2** | **1** | **1** | **1** | **2** | **1** | **1** | **0** | **0** | **0** | **1** | **1** | **1** | **1** |
| Learning | 62 | 64 | 65 | 66 | 67 | 68 | 69 | 70 | 71 | 72 | 73 | 74 | 75 | 76 | 77 |
|  | **3** | **5** | **5** | **2** | **4** | **5** | **5** | **2** | **2** | **3** | **2** | **4** | **5** | **4** | **2** |
|  | 0 | 1 | 0 | 0 | 2 | 0 | 0 | 0 | 1 | 1 | 0 | 0 | 0 | 0 | 0 |
|  | 1 | 1 | 0 | 0 | 0 | 0 | 0 | 0 | 0 | 0 | 0 | 0 | 1 | 0 | 0 |
| Cognitive  Flexibility | 62 | 64 | 65 | 66 | 67 | 68 | 69 | 70 | 71 | 72 | 73 | 74 | 75 | 76 | 77 |
|  | **4** | **5** | **5** | **2** | **6** | **5** | **5** | **2** | **3** | **3** | **2** | **4** | **6** | **4** | **2** |
|  | 0 | 1 | 0 | 0 | 0 | 0 | 0 | 0 | 0 | 1 | 0 | 0 | 0 | 0 | 0 |
|  | 0 | 1 | 0 | 0 | 0 | 0 | 0 | 0 | 0 | 0 | 0 | 0 | 0 | 0 | 0 |

Supplementary Table 8. Clustering with respect to age (>16) and subtest: Planning, Learning and Cognitive Flexibility (V).

| **Scale** | **Age** | |
| --- | --- | --- |
| Planning | 78 | 80 |
|  | **1** | **1** |
|  | 0 | 0 |
|  | **1** | **1** |
| Learning | 78 | 80 |
|  | **2** | **2** |
|  | 0 | 0 |
|  | 0 | 0 |
| Cognitive  Flexibility | 78 | 80 |
|  | **2** | **1** |
|  | 0 | 1 |
|  | 0 | 0 |

Supplementary Table 9. Sample adequacy means. Kaiser-Meyer-Olkin.

| **Variable** | **KMO** |
| --- | --- |
| Number of shifts correctly assigned in Part 1 | .92 |
| Number of shifts correctly assigned in Part 2 | .89 |
| Learning potential to identify whether the customer wears a neoprene suit | .93 |
| Learning potential when it comes to assign the right order to the customers | .88 |
| Number of total correct ice creams delivered correctly without looking at the recipe book on Part 1 rounds | .82 |
| Number of correct #1 ice creams delivered without looking at the recipe book in Part 1 rounds. | .83 |
| Number of correct #1 ice creams delivered without looking at the recipe book in Part 2. | .81 |
| Number of total correct ice creams delivered correctly without looking at the recipe book on Part 2 rounds | .84 |
| Learning potential in relation to making ice cream #1 correctly | .97 |
| Learning potential in terms of flexibility when making ice cream #4 in Part 2 (which was ice cream #1 in Part 1) | .79 |
| Number of perseverations when making the ice creams in Part 2 | .91 |
| Learning potential in terms of flexibility when making ice cream #1 in Part 2 (which is different from ice cream #1 in Part 1) | .85 |

Supplementary Table 10. Unexplained Variance.

| **variable** | **uniqueness** |
| --- | --- |
| Number of shifts correctly assigned in Part 1 | .21 |
| Number of shifts correctly assigned in Part 2 | .16 |
| Learning potential to identify whether the customer wears a neoprene suit | .53 |
| Learning potential when it comes to assign the right order to the customers | .16 |
| Number of total correct ice creams delivered correctly without looking at the recipe book on Part 1 rounds | .01 |
| Number of correct #1 ice creams delivered without looking at the recipe book in Part 1 rounds. | .04 |
| Number of correct #1 ice creams delivered without looking at the recipe book in Part 2. | .14 |
| Number of total correct ice creams delivered correctly without looking at the recipe book on Part 2 rounds | 0 |
| Learning potential in relation to making ice cream #1 correctly | .25 |
| Learning potential in terms of flexibility when making ice cream #4 in Part 2 (which was ice cream #1 in Part 1) | .36 |
| Number of perseverations when making the ice creams in Part 2 | .78 |
| Learning potential in terms of flexibility when making ice cream #1 in Part 2 (which is different from ice cream #1 in Part 1) | .37 |

Supplementary Table 11. Standard Abbreviations in the Ice Cream VR Test Report.

| Abbreviation (prefix-suffix) | Description |
| --- | --- |
| t. | Totals |
| s1. | Part or set 1 |
| s2. | Part or set 2 |
| r00. | Round or turn, and number. Ranges from 0 to 14  [0 is Training]. |
| .a00. | Avatar number. Ranks from 0 to 3. |
| .h. | Ice cream |
| ...turn. \| tu | Shift |
| .n | The variable stores a value that it returns. |

Supplementary Table 12. Clinical Report Key Variables.

| Variable | Description |
| --- | --- |
| s1_tu_total_correct_n | Number of shifts correctly assigned in Part 1 |
| s2_tu_total_correct_n | Number of shifts correctly assigned in Part 2 |
| R01:14_axx:axx_n_performance | Neoprene avatar criterion learning potential score Rounds 1 to 14 |
| R01:14_axx:axx_t_performance | Learning potential score avatar ticket criterion in Rounds 1 to 14 |
| s1_h_score_n | Number of total correct ice creams delivered correctly without looking at the recipe book on Part 1 rounds |
| s2_h_score_n | Number of total correct ice creams delivered correctly without looking at the recipe book on Part 2 rounds |
| R01:07_axx:axx_h1_performance | Learning potential score for ice cream #1 in Part 1 (Rounds 1 to 7) |
| R08:14_axx:axx_h4_performance | Learning Potential Score for Ice Cream #4 at Rounds 8 to 14 |
| R08:14_axx:axx_h1_performance | Learning potential score for ice cream #1 at Rounds 8 to 14 |
| s1_h1_score_n | Number of correct #1 ice creams delivered without looking at the recipe book in Part 1 rounds |
| s2_h1_score_n | Number of correct #1 ice creams delivered without looking at the recipe book in Part 2 |
| s2_h_persepress_n | Indicates that a #3 is made when a #1 should be made in Part 2 (same ice cream ingredients but different ice cream number in Part 2, indicative of perseveration). |
| rx.tu.total.correct.n | Indicates the number of times the order of clients has been correctly set based on the rules set by the instructions |
| rx.ax.turn.info.is.correct.n | Indicates if the given position in the queue is the correct one for that avatar in that turn |
| rx.h.rawscore.n | Number of ice cream delivered correctly, regardless of whether or not the reference is consulted in turn. |
| rx.ax.h.info.is.correct.n | Indicates whether an avatar has been given the correct ice cream in their round. |

Supplementary Table 13. Test reliability and internal consistency.

| **variable** | **easiness** | **discrimination** | **alpha2.5** | **alpha** | **alpha97.5** | **omega** |
| --- | --- | --- | --- | --- | --- | --- |
| r01.tu.total.correct.n | .46 | .80 | .92 | .93 | .93 | .95 |
| r02.tu.total.correct.n | .66 | .68 | .92 | .93 | .93 | .95 |
| r03.tu.total.correct.n | .53 | .97 | .92 | .93 | .93 | .95 |
| r04.tu.total.correct.n | .77 | .55 | .92 | .93 | .93 | .95 |
| r05.tu.total.correct.n | .79 | .51 | .92 | .93 | .93 | .95 |
| r06.tu.total.correct.n | .50 | .90 | .92 | .93 | .93 | .95 |
| r07.tu.total.correct.n | .72 | .73 | .92 | .93 | .93 | .95 |
| r08.tu.total.correct.n | .54 | .96 | .92 | .93 | .93 | .95 |
| r09.tu.total.correct.n | .63 | .85 | .92 | .93 | .93 | .95 |
| r10.tu.total.correct.n | .73 | .64 | .92 | .93 | .93 | .95 |
| r11.tu.total.correct.n | .68 | .63 | .92 | .93 | .93 | .95 |
| r12.tu.total.correct.n | .63 | .86 | .92 | .93 | .93 | .95 |
| r13.tu.total.correct.n | .62 | .86 | .92 | .93 | .93 | .95 |
| r14.tu.total.correct.n | .55 | .96 | .92 | .93 | .93 | .95 |
| r01.a00.turn.info.is.correct.n | .73 | .35 | .96 | .97 | .97 | .97 |
| r01.a01.turn.info.is.correct.n | .51 | .66 | .96 | .97 | .97 | .97 |
| r01.a02.turn.info.is.correct.n | .63 | .65 | .96 | .97 | .97 | .97 |
| r01.a03.turn.info.is.correct.n | .83 | .46 | .96 | .97 | .97 | .97 |
| r02.a00.turn.info.is.correct.n | .90 | .25 | .96 | .97 | .97 | .97 |
| r02.a01.turn.info.is.correct.n | .72 | .55 | .96 | .97 | .97 | .97 |
| r02.a02.turn.info.is.correct.n | .70 | .60 | .96 | .97 | .97 | .97 |
| r02.a03.turn.info.is.correct.n | .85 | .35 | .96 | .97 | .97 | .97 |
| r03.a00.turn.info.is.correct.n | .64 | .72 | .96 | .97 | .97 | .97 |
| r03.a01.turn.info.is.correct.n | .60 | .77 | .96 | .97 | .97 | .97 |
| r03.a02.turn.info.is.correct.n | .81 | .50 | .96 | .97 | .97 | .97 |
| r03.a03.turn.info.is.correct.n | .61 | .78 | .96 | .97 | .97 | .97 |
| r04.a00.turn.info.is.correct.n | .83 | .43 | .96 | .97 | .97 | .97 |
| r04.a01.turn.info.is.correct.n | .79 | .49 | .96 | .97 | .97 | .97 |
| r04.a02.turn.info.is.correct.n | .84 | .36 | .96 | .97 | .97 | .97 |
| r04.a03.turn.info.is.correct.n | .89 | .29 | .96 | .97 | .97 | .97 |
| r05.a00.turn.info.is.correct.n | .87 | .35 | .96 | .97 | .97 | .97 |
| r05.a01.turn.info.is.correct.n | .86 | .33 | .96 | .97 | .97 | .97 |
| r05.a02.turn.info.is.correct.n | .87 | .31 | .96 | .97 | .97 | .97 |
| r05.a03.turn.info.is.correct.n | .84 | .42 | .96 | .97 | .97 | .97 |
| r06.a00.turn.info.is.correct.n | .84 | .39 | .96 | .97 | .97 | .97 |
| r06.a01.turn.info.is.correct.n | .59 | .83 | .96 | .97 | .97 | .97 |
| r06.a02.turn.info.is.correct.n | .58 | .70 | .96 | .97 | .97 | .97 |
| r06.a03.turn.info.is.correct.n | .59 | .81 | .96 | .97 | .97 | .97 |
| r07.a00.turn.info.is.correct.n | .82 | .49 | .96 | .97 | .97 | .97 |
| r07.a01.turn.info.is.correct.n | .83 | .47 | .96 | .97 | .97 | .97 |
| r07.a02.turn.info.is.correct.n | .77 | .62 | .96 | .97 | .97 | .97 |
| r07.a03.turn.info.is.correct.n | .79 | .57 | .96 | .97 | .97 | .97 |
| r08.a00.turn.info.is.correct.n | .59 | .94 | .96 | .97 | .97 | .97 |
| r08.a01.turn.info.is.correct.n | .66 | .79 | .96 | .97 | .97 | .97 |
| r08.a02.turn.info.is.correct.n | .81 | .46 | .96 | .97 | .97 | .97 |
| r08.a03.turn.info.is.correct.n | .59 | .87 | .96 | .97 | .97 | .97 |
| r09.a00.turn.info.is.correct.n | .86 | .32 | .96 | .97 | .97 | .97 |
| r09.a01.turn.info.is.correct.n | .67 | .76 | .96 | .97 | .97 | .97 |
| r09.a02.turn.info.is.correct.n | .89 | .27 | .96 | .97 | .97 | .97 |
| r09.a03.turn.info.is.correct.n | .66 | .82 | .96 | .97 | .97 | .97 |
| r10.a00.turn.info.is.correct.n | .92 | .17 | .96 | .97 | .97 | .97 |
| r10.a01.turn.info.is.correct.n | .77 | .55 | .96 | .97 | .97 | .97 |
| r10.a02.turn.info.is.correct.n | .82 | .47 | .96 | .97 | .97 | .97 |
| r10.a03.turn.info.is.correct.n | .81 | .43 | .96 | .97 | .97 | .97 |
| r11.a00.turn.info.is.correct.n | .78 | .45 | .96 | .97 | .97 | .97 |
| r11.a01.turn.info.is.correct.n | .72 | .55 | .96 | .97 | .97 | .97 |
| r11.a02.turn.info.is.correct.n | .81 | .40 | .96 | .97 | .97 | .97 |
| r11.a03.turn.info.is.correct.n | .88 | .28 | .96 | .97 | .97 | .97 |
| r12.a00.turn.info.is.correct.n | .68 | .73 | .96 | .97 | .97 | .97 |
| r12.a01.turn.info.is.correct.n | .78 | .58 | .96 | .97 | .97 | .97 |
| r12.a02.turn.info.is.correct.n | .68 | .76 | .96 | .97 | .97 | .97 |
| r12.a03.turn.info.is.correct.n | .91 | .21 | .96 | .97 | .97 | .97 |
| r13.a00.turn.info.is.correct.n | .88 | .32 | .96 | .97 | .97 | .97 |
| r13.a01.turn.info.is.correct.n | .81 | .54 | .96 | .97 | .97 | .97 |
| r13.a02.turn.info.is.correct.n | .66 | .78 | .96 | .97 | .97 | .97 |
| r13.a03.turn.info.is.correct.n | .64 | .82 | .96 | .97 | .97 | .97 |
| r14.a00.turn.info.is.correct.n | .60 | .95 | .96 | .97 | .97 | .97 |
| r14.a01.turn.info.is.correct.n | .65 | .80 | .96 | .97 | .97 | .97 |
| r14.a02.turn.info.is.correct.n | .61 | .83 | .96 | .97 | .97 | .97 |
| r14.a03.turn.info.is.correct.n | .86 | .40 | .96 | .97 | .970 | .97 |
| r01.h.rawscore.n | .53 | .45 | .80 | .83 | .83 | .87 |
| r02.h.rawscore.n | .68 | .58 | .80 | .83 | .83 | .87 |
| r03.h.rawscore.n | .68 | .58 | .80 | .83 | .83 | .87 |
| r04.h.rawscore.n | .78 | .46 | .80 | .83 | .83 | .87 |
| r05.h.rawscore.n | .75 | .55 | .80 | .83 | .83 | .87 |
| r06.h.rawscore.n | .81 | .35 | .80 | .83 | .83 | .87 |
| r07.h.rawscore.n | .82 | .40 | .80 | .83 | .83 | .87 |
| r08.h.rawscore.n | .49 | .52 | .80 | .83 | .83 | .87 |
| r09.h.rawscore.n | .39 | .59 | .80 | .83 | .83 | .87 |
| r10.h.rawscore.n | 3.23 | 1.55 | .80 | .83 | .83 | .87 |
| r11.h.rawscore.n | .54 | .78 | .80 | .83 | .83 | .87 |
| r12.h.rawscore.n | .51 | .76 | .80 | .83 | .83 | .87 |
| r13.h.rawscore.n | .61 | .73 | .80 | .83 | .83 | .87 |
| r14.h.rawscore.n | .61 | .74 | .80 | .83 | .83 | .87 |
| r01.a00.h.info.is.correct.n | .78 | .38 | .92 | .93 | .93 | .94 |
| r01.a01.h.info.is.correct.n | .80 | .28 | .92 | .93 | .93 | .94 |
| r01.a02.h.info.is.correct.n | .75 | .41 | .92 | .93 | .93 | .94 |
| r01.a03.h.info.is.correct.n | .78 | .28 | .92 | .93 | .93 | .94 |
| r02.a00.h.info.is.correct.n | .85 | .29 | .92 | .93 | .93 | .94 |
| r02.a01.h.info.is.correct.n | .85 | .36 | .92 | .93 | .93 | .94 |
| r02.a02.h.info.is.correct.n | .86 | .33 | .92 | .93 | .93 | .94 |
| r02.a03.h.info.is.correct.n | .86 | .33 | .92 | .93 | .93 | .94 |
| r03.a00.h.info.is.correct.n | .87 | .28 | .92 | .93 | .93 | .94 |
| r03.a01.h.info.is.correct.n | .84 | .37 | .92 | .93 | .93 | .94 |
| r03.a02.h.info.is.correct.n | .86 | .30 | .92 | .93 | .93 | .94 |
| r03.a03.h.info.is.correct.n | .85 | .29 | .92 | .93 | .93 | .94 |
| r04.a00.h.info.is.correct.n | .87 | .32 | .92 | .93 | .93 | .94 |
| r04.a01.h.info.is.correct.n | .91 | .24 | .92 | .93 | .93 | .94 |
| r04.a02.h.info.is.correct.n | .91 | .19 | .92 | .93 | .93 | .94 |
| r04.a03.h.info.is.correct.n | .91 | .20 | .92 | .93 | .93 | .94 |
| r05.a00.h.info.is.correct.n | .91 | .22 | .92 | .93 | .93 | .94 |
| r05.a01.h.info.is.correct.n | .89 | .27 | .92 | .93 | .93 | .94 |
| r05.a02.h.info.is.correct.n | .93 | .17 | .92 | .93 | .93 | .94 |
| r05.a03.h.info.is.correct.n | .86 | .31 | .92 | .93 | .93 | .94 |
| r06.a00.h.info.is.correct.n | .89 | .19 | .92 | .93 | .93 | .94 |
| r06.a01.h.info.is.correct.n | .93 | .14 | .92 | .93 | .93 | .94 |
| r06.a02.h.info.is.correct.n | .93 | .17 | .92 | .93 | .93 | .94 |
| r06.a03.h.info.is.correct.n | .93 | .17 | .92 | .93 | .93 | .94 |
| r07.a00.h.info.is.correct.n | .91 | .22 | .92 | .93 | .93 | .94 |
| r07.a01.h.info.is.correct.n | .92 | .16 | .92 | .93 | .93 | .94 |
| r07.a02.h.info.is.correct.n | .92 | .19 | .92 | .93 | .93 | .94 |
| r07.a03.h.info.is.correct.n | .94 | .17 | .92 | .93 | .93 | .94 |
| r08.a00.h.info.is.correct.n | .75 | .37 | .92 | .93 | .93 | .94 |
| r08.a01.h.info.is.correct.n | .72 | .32 | .92 | .93 | .93 | .94 |
| r08.a02.h.info.is.correct.n | .84 | .36 | .92 | .93 | .93 | .94 |
| r08.a03.h.info.is.correct.n | .78 | .37 | .92 | .93 | .93 | .94 |
| r09.a00.h.info.is.correct.n | .63 | .36 | .92 | .93 | .93 | .94 |
| r09.a01.h.info.is.correct.n | .80 | .41 | .92 | .93 | .93 | .94 |
| r09.a02.h.info.is.correct.n | .73 | .45 | .92 | .93 | .93 | .94 |
| r09.a03.h.info.is.correct.n | .69 | .50 | .92 | .93 | .93 | .94 |
| r10.a00.h.info.is.correct.n | .85 | .29 | .92 | .93 | .93 | .94 |
| r10.a01.h.info.is.correct.n | .72 | .52 | .92 | .93 | .93 | .94 |
| r10.a02.h.info.is.correct.n | .75 | .44 | .92 | .93 | .93 | .94 |
| r10.a03.h.info.is.correct.n | .90 | .21 | .92 | .93 | .93 | .94 |
| r11.a00.h.info.is.correct.n | .87 | .30 | .92 | .93 | .93 | .94 |
| r11.a01.h.info.is.correct.n | .83 | .36 | .92 | .93 | .93 | .94 |
| r11.a02.h.info.is.correct.n | .74 | .49 | .92 | .93 | .93 | .94 |
| r11.a03.h.info.is.correct.n | .79 | .42 | .92 | .93 | .93 | .94 |
| r12.a00.h.info.is.correct.n | .72 | .50 | .92 | .93 | .93 | .94 |
| r12.a01.h.info.is.correct.n | .76 | .48 | .92 | .93 | .93 | .94 |
| r12.a02.h.info.is.correct.n | .87 | .31 | .92 | .93 | .93 | .94 |
| r12.a03.h.info.is.correct.n | .77 | .45 | .92 | .93 | .93 | .94 |
| r13.a00.h.info.is.correct.n | .87 | .29 | .92 | .93 | .93 | .94 |
| r13.a01.h.info.is.correct.n | .76 | .48 | .92 | .93 | .93 | .94 |
| r13.a02.h.info.is.correct.n | .86 | .31 | .92 | .93 | .93 | .94 |
| r13.a03.h.info.is.correct.n | .86 | .28 | .92 | .93 | .93 | .94 |
| r14.a00.h.info.is.correct.n | .80 | .43 | .92 | .93 | .93 | .94 |
| r14.a01.h.info.is.correct.n | .85 | .34 | .92 | .93 | .93 | .94 |
| r14.a02.h.info.is.correct.n | .89 | .28 | .92 | .93 | .93 | .94 |
| r14.a03.h.info.is.correct.n | .78 | .46 | .92 | .93 | .93 | .94 |
